# Wavelet-domain elastic net for clustering on genomes strains

**DOI:** 10.1590/1678-4685-GMB-2018-0035

**Published:** 2018-11-29

**Authors:** Leila Maria Ferreira, Thelma Sáfadi, Juliano Lino Ferreira

**Affiliations:** ^1^ Programa de Pós-Graduação em Estatística e Experimentação Agropecuária, Departamento de Estatística, Universidade Federal de Lavras (UFLA), Lavras, MG, Brazil; ^2^ Departamento de Estatística, Universidade Federal de Lavras (UFLA), Lavras, MG, Brazil; ^3^ Empresa Brasileira de Pesquisa Agropecuária (Embrapa) Pecuária Sul. Bagé, RS, Brazil

**Keywords:** Elastic net, genome, GC-content, cluster analysis, wavelet transform

## Abstract

We propose to evaluate genome similarity by combining discrete non-decimated wavelet transform (NDWT) and elastic net. The wavelets represent a signal with levels of detail, that is, hidden components are detected by means of the decomposition of this signal, where each level provides a different characteristic. The main feature of the elastic net is the grouping of correlated variables where the number of predictors is greater than the number of observations. The combination of these two methodologies applied in the clustering analysis of the *Mycobacterium tuberculosis* genome strains proved very effective, being able to identify clusters at each level of decomposition.

## Introduction

*Mycobacterium tuberculosis* (MTB), also called Koch bacillus, is a species of the pathogenic bacterium of the genus *Mycobacterium* and the causative agent of most cases of tuberculosis (TB) (Taylor *et al.*, 2003). TB is the ninth leading cause of death worldwide and the leading cause of death from a single infectious agent, ranking above HIV/AIDS. In 2016, there were an estimated 1.3 million TB deaths among HIV-negative people (down from 1.7 million in 2000) and an additional 374,000 deaths among HIV-positive people. An estimated 10.4 million people fell ill with TB in 2016: 90% were adults, 65% were male, 10% were people living with HIV (74% in Africa), and 56% were in five countries: India, Indonesia, China, the Philippines, and Pakistan. Drug-resistant TB is a continuing threat. In 2016, there were 600,000 new cases with TB resistance to rifampicin, the most effective first-line drug, of which 490,000 had multidrug-resistant TB (MDR). Almost half (47%) of these cases were in India, China, and the Russian Federation (WHO, 2017).

By the end of 2016, extensively drug-resistant TB (XDR) had been reported by 123 WHO member states. Of these, 91 countries and five territories reported representative data from continuous surveillance or surveys regarding the proportion of MDR cases that had XDR. Combining their data, the average proportion of MDR cases with XDR was 6.2% (95% CI: 3.6–9.5%) (WHO, 2017)

Several studies are underway regarding the study of MTB strains in order to detect the mutations that the strains suffer when becoming drug resistant. Boehme *et al.* (2010) studied an automated molecular test for MTB resistance to rifampin (RIF) in patients with suspected drug-sensitive or multidrug-resistant pulmonary tuberculosis. Perdigão *et al.* (2010) performed a study to characterize the genetic changes associated with the high number of XDR TB that threatens the control of TB worldwide. Zhou *et al.* (2017) investigated the association between genotype and drug resistance profiles of MTB strains circulating in China in a national drug resistance survey. Müller *et al.* (2013) studied programmatically selected multidrug-resistant strains drive and the emergence of extensively drug-resistant TB in South Africa. Smith *et al.* (2014) investigated the reduced virulence of an XDR outbreak strain of MTB in a murine model. Other related studies were done by Iwamoto *et al.* (2012), Sandegren *et al.* (2011), Buu *et al.* (2012), and Treviño *et al.* (2015).

In order to extract information regarding the genome of MTB, one of the techniques used is the GC-content. This is an important parameter of bacterial genomes used to scan the basic composition of the genome, as well as to understand the evolution of the coded sequence. Hildebrand *et al.* (2010) showed that the GC-content is highly correlated to genomic GC-content, that is, there is selection on genomic base composition in many bacteria.

The use of wavelet analysis in genomic data has been growing a growing field. One of the features of this analysis is the extraction of characteristics that are hidden, thus increasing the precision of the results. Conceptually, the wavelet transform is a technique for seeing and representing a signal. This signal is decomposed in resolution levels, where each level adds details. Mathematically, it is represented by a function oscillating in time or space. The method has sliding windows that expand or compress to capture low and high frequency signals, respectively (Percival and Walden, 2000). Its origin occurred in the field of seismic study to describe the disturbances arising from a seismic impulse (Morlet *et al.*, 1982).

Among the wavelet techniques, we used the discrete non-decimated wavelet transform (NDWT), which has as its main characteristic that it can work with any size of signals/sequences. In this technique, the coefficients are translation invariants, that is, the choice of origin is irrelevant, since all the observations are used in the analysis, a situation that does not occur in the discrete decimated wavelet transform (DWT). Discrete wavelet transforms have been used to identify gene locations in genomic sequences (Ning *et al.*, 2003), identifying long-range correlations, locating periodicities in DNA sequences (Vannucci and Liò, 2001), and for analysis of G+C patterns (Dodin *et al.*, 2000).

The NDWT method can be used in any genome type, increasing the speed of the analysis, which is processed almost in real time. Bao and Yuan (2015) created a wavelet-based feature vector (WFV) model that outperformed the other models in terms of both the clustering results and the running time, confirming that wavelets are an efficient method in the analysis of DNA sequences.

The clustering analysis that has been worked with genomic data is the elastic net, which is the regular regression method that linearly combines the L_1_ and L_2_ penalties of the LASSO and Ridge regression methods. The main feature of this method is the grouping of correlated variables where the number of predictors is greater than the number of observations. Elastic net employs a grouping effect, in which strongly correlated predictors tend to be in or out of the model. Sáfadi (2017) showed that the wavelet-domain elastic net methodology was effective for clustering of time series data, that is, the interaction of wavelets with elastic net is an efficient method of grouping. Another characteristic of the method is the speed with which the analyses are processed. Mol *et al.* (2009) proved that there exists a particular “elastic net representation” of the regression function such that, if the number of data increases, the elastic net estimator is consistent not only for prediction but also for variable/feature selection, demonstrating the adaptive capacity of the elastic net. Cho *et al.* (2009) proposed a simple stepwise procedure that identifies disease-causing SNPs simultaneously by employing elastic net regularization, a variable selection method that allows addressing multicollinearity in the study of rheumatoid arthritis, showing the efficiency of genetic data interaction with elastic net. The studies of Waldmann *et al.* (2013), Hughey and Butte (2015), Ayers and Cordell (2010), Ogutu *et al.* (2012), and Furqan and Siyal (2016) also show the significant relationship between genetic data interaction and elastic net.

In this work, the discrete non-decimated wavelet transform was applied to GC-content sequences; the detailed level coefficients are used to study similarities of MTB genome strains through elastic net methodology. The visualization of the graphs obtained with the elastic net allowed identifying the groupings of similar strains. The proposed methodology was applied to ten MTB sequences, with two being 2 drug-resistant, 6 six drug-susceptible, one multi drug-resistant and one extensively drug-resistant.

## Material and Methods

In the analyses, the free software R (R Core Team, 2017) was used.

Table 1 shows the description of each strain of the MTB genome, obtained from the National Center for Biotechnology Information (NCBI, 2017). The methodology used was as follows:

**Table 1:** Descriptions of the *Mycobacterium tuberculosis* strains.

Sequences	Descriptions of the strains
Seq1_DS	Strain was isolated in Russia belonging to the AI family (according to RFLP genotyping) and it is sensitive to all common drugs used in the treatment of tuberculosis.
Seq2_DS	Susceptible strain representing the largest portion of tuberculosis isolates recovered during an epidemic in the Western Cape of South Africa.
Seq3_DS	Susceptible strain belonging to the Beijing family, sequenced for comparative genomic studies.
Seq4_DR	Resistant strain isolated in 2004, referring to a patient with secondary pulmonary tuberculosis, sequenced for comparative genomic studies.
Seq5_DR	Drug-resistant strain, having an accelerated rate of transmission between humans under agglomeration conditions.
Seq6_MDR	Strain from a single patient in KwaZulu-Natal, South Africa.
Seq7_XDR	Strain from a single patient in KwaZulu-Natal, South Africa.
Seq8_DS	Susceptible strain used for comparative genomic studies.
Seq9_DS	Susceptible strain derived from the original human lung H37, isolated in 1934. It has been widely used all over the world in biomedical research. Unlike some clinical isolates, it retains total virulence in animals with tuberculosis and is susceptible to drugs and receptive to genetic manipulation.
Seq10_DS	A virulent susceptible strain derived from its virulent parent strain H37 (isolated from a 19-year-old male patient with chronic pulmonary tuberculosis, named Edward R. Baldwin in 1905). This strain was obtained through an aging and dissociation process of an *in vitro* culture in 1935.

1. The GC-content of all the sequences was evaluated using a sliding window of 10,000 base pairs (bp).

The GC-content is an important parameter of bacterial genomes used to scan the basic composition of the genome as well as to understand the evolution of the coded sequence. Generally, the CG-content ranges from 25 to 75% in bacterial genomes (Mann and Chen, 2010). In the mammalian genome, approximately 50% of all genes are controlled by promoters with high GC-content. Chang *et al.* (2015) examined a method for stable quantification of such GC-rich DNA sequences.

For each genome sequence, the GC-content is calculated as the ratio of the sum of G and C bases divided by the sum of the A, G, C and T bases (Equation 1):

(1) $$GCcontent=\frac{nG+nC}{nA+nG+nC+nT}$$

where nA, nG, nC, and nT are the number of A, G, C and T nucleotide bases, respectively, in a sequence. The GC-content can also be calculated for a part of the sequence using the window technique, wherein the GC-content is calculated for a fixed length of a specific window of the sequence. The determination of GC-content ratio helps in identifying gene-rich regions of the genome (Saini and Dewan, 2016). Theses gene-rich regions provide significant biological information about the genome. Cheng *et al.* (2016) and Wei *et al.* (2016) worked with high GC-content aiming to develop new molecular markers, highlighting the importance of working with gene-rich regions.

2. The sequences were decomposed using a discrete non-decimated wavelet transform. We used the Daubechies wavelet (4 null moments) with 5 levels of decomposition.

A wavelet function is the interpretation of a short wave with rapid increase and decrease. The theory is based on the representation of functions in different scales and resolutions (time-scale), being considered one of its main characteristics (Daubechies, 1992).

In the analysis of wavelets, the oscillating window is called the mother wavelet. There are arbitrary translations and dilations, and thus the mother wavelet generates other wavelets (Hernadez and Weiss, 1996).

By definition: a wavelet is a function $\text{ψ}(x)\in {\text{L}}^{2}(\mathbb{R})$, such that the function family is given by Equation 2:

(2) $${\text{ψ}}_{j,k}=2^{-\frac{j}{2}}{\text{ψ(2}}^{-j}x-k),$$

where *j* and *k* are arbitrary integers on an orthonormal basis in Hilbert space ${\text{L}}^{2}(\mathbb{R})$ (Wojtaszczyk, 1997).

The characteristic of the discrete non-decimated wavelet transform (NDWT) is to keep the same amount of data in the even and odd decimations on each scale and continue to do the so on each subsequent scale. The coefficients are translational invariants, that is, the circular displacement of the data is reflected in the same direction of the coefficients. Another feature is the ability to handle data of arbitrary size that does not require the sample size to be a power of two, which is what occurs in the discrete decimated wavelet transform (Nason, 2008). The main advantage of this method is associated with zero-pass filters, which means that it operates circularly to the data allowing functionalities at different scales to be aligned with the sequence of the original data (Vannucci and Liò, 2001).

Percival and Walden (2000) highlight that the NDWT method can also be used to form a multiresolution analysis (MRA). The approximation coefficients and coefficients of details of MRA are such that circularly shifting the time-series by any amount will circularly shit each approximation coefficients and coefficients of details by a corresponding amount. The NDWT method is computed O(N log_2_ N) using multiplications.

The Daubechies wavelet is a family of orthogonal wavelets that define a discrete wavelet transformation, characterized by a maximum number of null moments (degree of smoothing) for some given support. With each wavelet type of this class, there is a scaling function (called father wavelet), which generates an orthogonal multiresolution analysis.

According to Daubechies (1992), for each integer *r*, the orthonormal basis for ${\text{L}}^{2}(\mathbb{R})$ is defined by Equation 3:

(3) $${\text{ψ}}_{r,j,k}=2^{-\frac{j}{2}}{\text{ψ}}_{r}{\text{(2}}^{-j}x-k),\;\;\;\;\;j,k\in \mathbb{Z}$$

in which the function ${\text{ψ}}_{r}(x)$ in ${\text{L}}^{2}(\mathbb{R})$ has the property that ${\text{ψ}}_{r}(x-k)|k\in \mathbb{Z}$ is an orthonormal sequential basis in ${\text{L}}^{2}(\mathbb{R})$. Here, *j* is the scale index, *k* is the translation index, and *r* is the filtering index.

3. The elastic net methodology was used at each level of decomposition, aiming at the identification of similar sequences.

According to Zou and Hastie (2005), given a set of data with *n* observations and *p* predictors, considering ***y*** = (*y*_1_ ,…, *y_n_*)^*T*^ the response **X** = (**x_1_** |…|**x_p_**) the matrix model, where in ***x_j_*** = (*x*_1*j*_ ,…, *x_nj_*)^*T*^, *j* = 1,…, *p*, are the predictors, and considering that the response is centralized and the predictors are standardized, that is, correlated,

(4) $$\sum_{i=1}^{n}y_{i}=0\sum_{i=1}^{n}x_{ij}=0\text{and}\sum_{i=1}^{n}x_{ij}^{2}=1\;\;\;\;\;\text{for}\;j=1,\ldots ,\text{p}.$$

For any λ_1_ e λ_2_ fixed and non-negative, the elastic net criterion is defined as:

(5) $$\text{L(}\lambda_{\text{1}},\lambda_{\text{2}},\text{β)=}{\left|y-X\text{β}\right|}^{2}\lambda_{2}{\left|\text{β}\right|}^{2}+\lambda_{1}{\left|\text{β}\right|}^{2},$$

wherein

$${\left|\text{β}\right|}^{2}=\sum_{\text{j=1}}^{\text{p}}{\text{β}}_{j}^{2}$$

$${\left|\text{β}\right|}_{1}=\sum_{\text{j=1}}^{\text{p}}\left|{\text{β}}_{j}\right|.$$

The elastic net estimator $\hat{\beta }$ is the minimizer of Equation 5

(6) $$\hat{\text{β}}=\arg\;\underset{\text{β}}{\min}\left\{\lambda_{1},\lambda_{2},\text{β}\right\}.$$

This procedure can be seen as a penalized least squares method. Let $\alpha =\frac{\lambda_{2}}{\lambda_{1}+\lambda_{2}}$, so solving $\hat{\beta }$ in equation 5 is equivalent to the optimized problem

(7) $$\begin{array}{l} \hat{\text{β}}=\arg\;\underset{\text{β}}{\min}{\left|y-X\text{β}\right|}^{2},\;\text{subject}\;\text{to}\;(1-\alpha ){\left|\text{β}\right|}_{1}+\\ +\alpha {\left|\text{β}\right|}^{2}\leq t\;\text{for}\;\text{some}\;t. \end{array}$$

**Figure 1:**
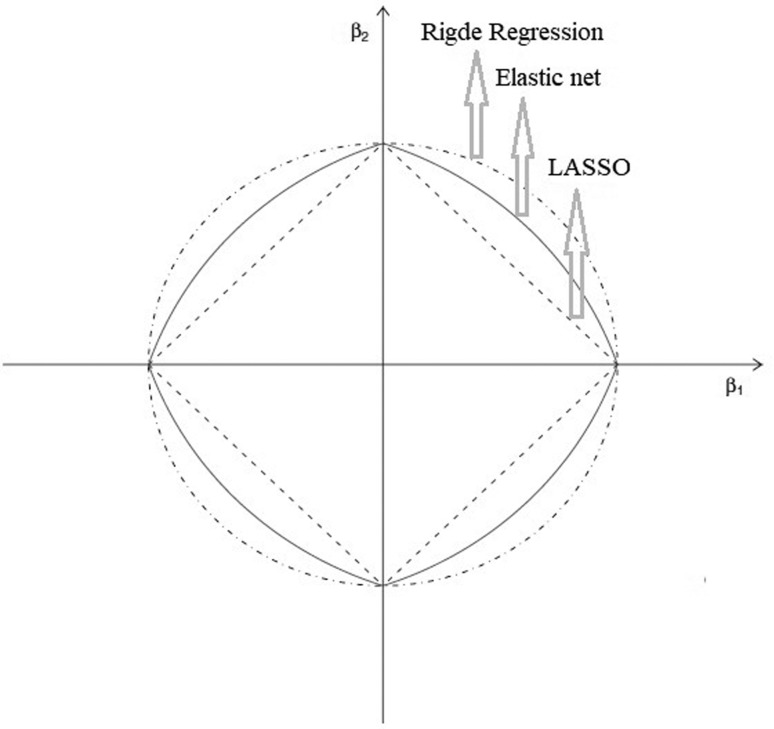
Geometry of the penalties. Source: Zou and Hastie (2005).

The function $(1-\alpha ){\left|\text{β}\right|}_{1}+\alpha {\left|\text{β}\right|}^{2}$ is called elastic net penalty and is a convex combination between the penalties that define the LASSO and Rigde estimation, respectively. When α = 1 the elastic net becomes a simple Ridge regression. When α = 0, we have the LASSO penalty, which is convex but not strictly convex. When α = 0.5 we have elastic net penalty. These arguments can be seen in Figure 1.

The Ridge regression estimator (keeping all predictors) is:

(8) $$\hat{\text{β}}=\arg\;\min\left\{\sum_{i=1}^{n}{\left(Y_{i}-\text{Xβ}\right)}^{2}+\lambda \sum_{j}{\text{β}}_{j}^{2}\right\}.$$

The LASSO estimator (keeping the most significant predictors and removing the others) is:

(9) $$\hat{\text{β}}\text{=arg min}\left\{\sum_{i=1}^{n}{\left(Y_{i}-\text{Xβ}\right)}^{2}+\text{μ}\sum_{j}\left|\beta_{j}\right|\right\}.$$

The elastic net is a combination of Ridge regression and LASSO; its estimator is given by

(10) $$\begin{array}{l} \hat{\text{β}}\text{=arg min}\{\sum_{i=1}^{n}{\left(Y_{i}-\text{Xβ}\right)}^{2}\\ +\lambda \left(\left(1-\alpha \right)\sum_{j}\left|{\text{β}}_{j}\right|+\alpha \sum_{j}{\text{β}}_{j}^{2}\right)\}. \end{array}$$

## Results and Discussion

Table 2 contains the information for each sequence of strains of the MTB genome obtained from NCBI. Note that the GC-content total rate values are very close, indicating that there are no differences between the sequences.

**Table 2:** Description of the *Mycobacterium tuberculosis* genome.

Sequence number	NCBI Access number	Resistance type	Total Rate of GC-content	Infraspecific name
Seq1	CP002992.1	DS	0.6560	CTRI-2
Seq2	CP000717.1	DS	0.6562	F11
Seq3	CP001641.1	DS	0.6561	CCDC5079
Seq4	CP001642.1	DR	0.6559	CCDC5180
Seq5	CP001664.1	DR	0.6563	str. Haarlem
Seq6	CP001658.1	MDR	0.6561	KZN 1435
Seq7	CP001976.1	XDR	0.6561	KZN 605
Seq8	CP002884.1	DS	0.6561	CCDC5079
Seq9	AL123456.3	DS	0.6561	H37Rv
Seq10	CP000611.1	DS	0.6561	H37Ra

DS = drug susceptible; DR = drug resistant; MDR = multidrug resistant; XDR = extensively drug resistant.

**Figure 2:**
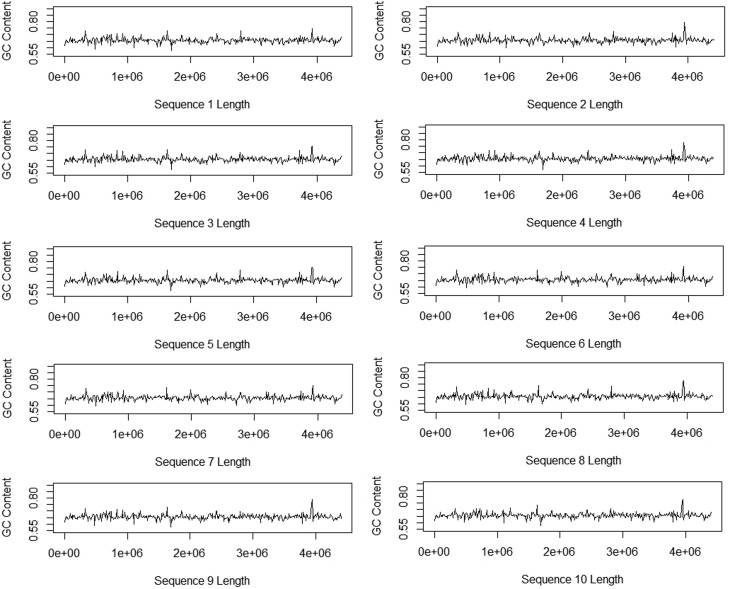
GC-content sequence sign (10,000 bp window) of MTB strains.

Figure 2 shows the size and signal behavior visualization of each GC-content sequence. Note that the sequences show practically the same behavior. The *x*-axis shows the amount of nucleotides of each sequence.

We applied the proposed methodology to the GC-content sequences. After comparing locality and smoothness of the decomposing wavelet, the Daubechies with 4 vanishing moments, db4, to the non-decimated wavelet decomposition was selected. The coefficients at each multiresolution level are denoted by d1, d2, d3, d4, d5, and s5 with d1 being the level of the finest detail and s5 the smoothest level.

**Figure 3:**
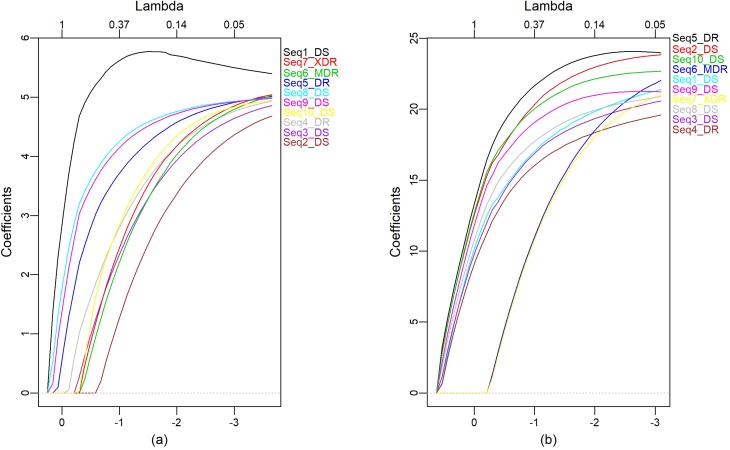
Elastic net for: (a) signals of the GC-content sequences, (b) s5 coefficients.

First, the elastic net was applied on the GC-content sequence and on the smooth level coefficients (s5, Figure 3), which revealed three groups (Figure 3a). The first group was composed of three members, the second of five, whereas the third one contained only Seq2_DS. Considering the smooth level coefficients, (Figure 3b) the first group is made up by the sequences Seq6_MDR and Seq7_XDR and the others are in the second group.

Comparing the formation of the groups without NDWT with the groups formed with NDWT, referring to the smooth level of decomposition, we found that in the second group formed without NDWT the elastic net failed to distinguish XDR and MDR strains from DS strains. This is a contradictory situation, since belong completely different strains. In the NDWT, referring to the smooth level of decomposition, this separation occurs very clearly, showing that the XDR and MDR strains are different in relation the other strains analyzed and between them are similar.

**Figure 4:**
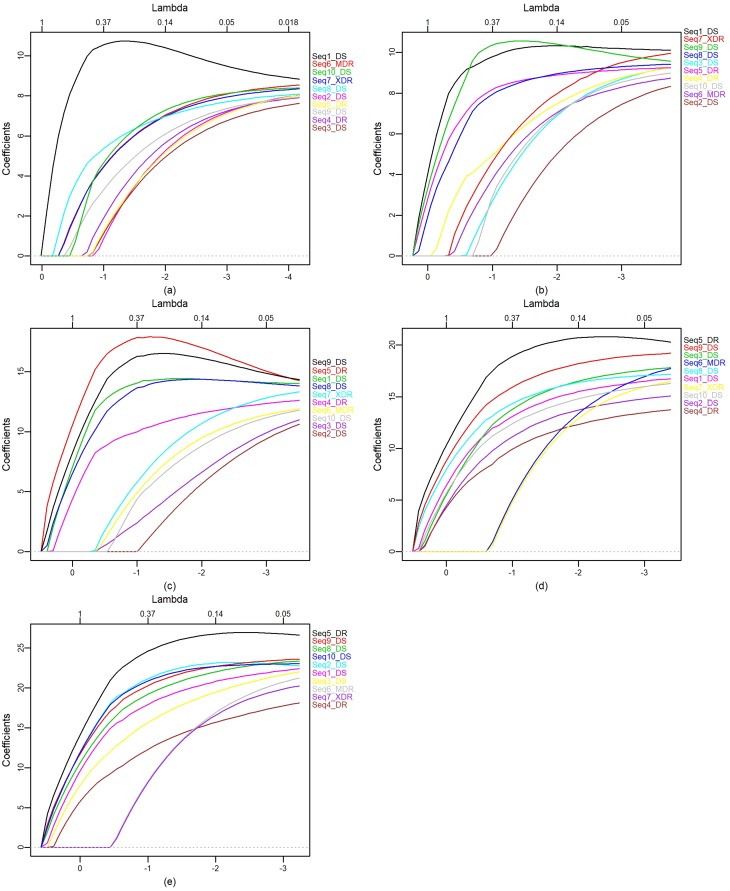
Elastic net for: (a) d1, (b) d2, (c) d3, (d) d4, and (e) d5 coefficients.

Considering each level of detail, the elastic net was applied to d1 to d5 coefficients. Figure 4 shows the elastic net plots on each level. We summarized the clustering observed in Table 3.

**Table 3:** Formation of the groups at each level of decomposition.

Levels	Groups				
	1	2	3	4	5
1	DS{1}	DS{2, 3} DR{4, 5}	DS{8, 9, 10}{6-MDR, 7-XDR}		
2	DS{2}	DS{3, 10}	DR{4}	{6-MDR, 7-XDR}	DS{1, 8, 9} DR{5}
3	DS{2}	DS{3, 10} {6-MDR, 7-XDR}	DS{1, 8, 9} DR{4, 5}		
4	{6-MDR, 7-XDR}	DS{1, 2, 3, 8, 9, 10} DR{4, 5}			
5	{6-MDR, 7-XDR}	DS{1, 2, 3, 8, 9, 10} DR{4, 5}			

Table 3 shows that the 6-MDR and 7-XDR sequences were pooled at all levels of detail. These strains correspond to a single patient in KwaZulu-Natal, South Africa. At level 1, the highlight is for the 1-DS sequence that alone forms a group; this strain was isolated in Russia from the AI family (according to RFLP genotyping), and was sensitive to all common drugs used in the treatment of TB. For levels 2 and 3, the sequence 2-DS formed a group; this is a susceptible strain representing the largest portion of TB isolates from patients recovered during an epidemic in the Western Cape region of South Africa. Level 2 also highlights the 4-DR sequence, which is a resistant strain isolated in 2004, referring to a patient with secondary pulmonary TB, sequenced for comparative genomic studies.

The 5-DR sequence corresponds to a drug-resistant strain, with an accelerated rate of transmission between humans under agglomeration conditions. The 8-DS sequence is a susceptible strain used for comparative genomic studies. The 9-DS sequence is a susceptible strain derived from the original human lung H37, isolated in 1934. It has been widely used all over the world in biomedical research. Unlike some clinical isolates, it retains total virulence in animals with TB and is susceptible to drugs and receptive to genetic manipulation. These sequences appear grouped at all levels, except for the first detail level.

In addition, the DS (3 and 10) sequences appear grouped at all levels, except for the first level of detail. The sequence 3-DS is a susceptible strain belonging to a Beijing family, sequenced for comparative genomic studies, and the 10-DS sequence is an avirulent susceptible strain derived from its virulent parent strain H37 (isolated in 1905 from a 19-year-old male patient named Edward R. Baldwin who had chronic pulmonary TB). This strain was obtained in 1935 through an aging and dissociation process of an *in vitro* culture.

Concerning group formation, at levels 4 and 5 these groups were the same, forming two groups. At level 2, the largest number of groups were formed, totaling five. At this level, a larger specification of the groups occurs, with two strains isolated.

Saini and Dewan (2016), based on the calculation of the energy of wavelet decomposition coefficients of complete genomic sequences, showed that the genomic sequences of MTB could be grouped only into two groups. The first group with DS and DR sequences (lower energy) and the second group with MDR and XDR sequences (highest energy). Ferreira *el al*. (2017), considering the energy at each level of detail, were able to identify more than two groups, as particularities of 1 (DS), 3 (DS), and 4 (DR) sequences were detected with the proposed methodology.

## Conclusions

The combination of the NDWT and elastic net methodologies, applied in the analysis of clustering of the *Mycobacterium tuberculosis* genome strains, proved very effective. Through this analysis, it was possible to see group formation at each level of decomposition.
